# Continuous intake of quercetin-rich onion powder may improve emotion but not regional cerebral blood flow in subjects with cognitive impairment

**DOI:** 10.1016/j.heliyon.2023.e18401

**Published:** 2023-07-19

**Authors:** Yuichi Hayashi, Fuminori Hyodo, Tana, Kiyomi Nakagawa, Takuma Ishihara, Masayuki Matsuo, Takayoshi Shimohata, Jun Nishihira, Masuko Kobori, Toshiyuki Nakagawa

**Affiliations:** a Department of Neurobiology, Gifu University Graduate School of Medicine, Gifu, Japan; b Department of Neurology, Gifu University Graduate School of Medicine, Gifu, Japan; c Department of Radiology, Gifu University Graduate School of Medicine, Gifu, Japan; d Innovative and Clinical Research Promotion Center, Gifu University Hospital, Gifu, Japan; e Department of Medical Management and Informatics, Hokkaido Information University, Hokkaido, Japan; f Institute of Food Research, National Agriculture and Food Research Organization, Ibaraki, Japan; g Institute for Advanced Study Gifu University, Gifu, Japan; h Department of Nursing, University of Tokyo Health Science, Tokyo, Japan

**Keywords:** Behavioral and psychological symptoms of dementia, Alzheimer's disease, Mild cognitive impairment, Depression, Redox

## Abstract

Depression in later life is associated with dementia. Changes in motivated behavior are an important mechanism contributing to dysfunctional cognitive control in depression. Although continuous intake of quercetin-rich onion suppresses cognitive decline in aged people by improving their emotional condition, the effect of quercetin-rich onion on emotional condition in people living with cognitive impairment remains unclear. In this randomized, double-blind, placebo-controlled study of subjects with cognitive impairment, we found that subjects wrote more adjectives and adverbs per sentence on the Mini-Mental State Examination after intake of quercetin-rich onion powder than before intake, although regional cerebral blood flow on n-isopropyl-4-[123]iodoamphetamine hydrochloride single-photon emission computed tomography was not changed. In the EPM, mice that had received a quercetin-supplemented chow diet made a significantly increased number of exploratory head dips from the open arms of the maze. Moreover, the 3-methoxycarbonyl-2,2,5,5-tetramethyl-pyrrolidine-1-oxyl decay rate, reflecting redox activity, was increased in mice fed a quercetin-added diet. These results indicate that quercetin-rich onion may affect motivated behavior in subjects with cognitive impairment, for whom quercetin intake may preserve redox homeostasis in the brain.

## 1 Introduction

The number of people living with dementia has risen to approximately 55 million worldwide in 2019 [1], and this number is projected to increase to 152 million in 2050 [2]. Nonetheless, age-specific incidence rates of dementia are declining in some countries [3], probably due to lifestyle changes such as exercise, weight control and dietary interventions [4]. In Japan, the prevalence of dementia even after controlling for the confounding effects of aging increased in 2012 compared with two decades ago, which may be related to the increase in diabetes [5]. There are several modifiable risk factors whose elimination could prevent or delay the onset of dementia; these risk factors include obesity, hypertension, and depression [4]. Depression, especially in later life, is associated with dementia [6], and the prevalence of depression is 32% in people with mild cognitive impairment (MCI) [7]. Depression impairs attention, memory, and cognitive control. Changes in motivated behavior, such as reward anticipation, are an important mechanism contributing to dysfunctional cognitive control in depression, suggesting the connection of control deficits between motivation and cognition [8]. Adult hippocampal neurogenesis (AHN) in humans occurs in the subgranular zone of the dentate gyrus. Recently, several reports have suggested that increasing AHN suppresses depression and anxiety-like behavior [[9], [10], [11], [12]] and improves memory [9] in mice.

In animal models of several neurodegenerative conditions, such as Alzheimer's disease (AD), Parkinson's disease, and Huntington's disease, quercetin exerts positive effects through several mechanisms of action, including anti-inflammatory, pro-neurotrophic, and antioxidant mechanisms [13] as well as the integrated stress response [14]. Interestingly, quercetin alleviates depression-like behavior [15] by stimulating AHN via BDNF expression in mice after chronic unpredictable mild stress exposure [16]. We previously showed that the memory recall scores of AD patients on the Revised Hasegawa Dementia Scale (HDS-R) were increased by the intake of quercetin-rich onion powder containing 80 mg aglycone daily for four weeks [17]. Recently, Nishihira et al. demonstrated that a clinical trial of 24-week continuous intake of quercetin-rich onion (50 mg aglycone) suppresses age-related cognitive decline, possibly by improving the users' emotional condition [18]. However, it remains to be determined whether daily intake of quercetin-rich onion powder (50 mg aglycone per day) affects the emotional condition of people living with cognitive impairment. We conducted a randomized, double-blind, placebo-controlled study of the continuous intake of quercetin-rich onion powder and placebo onion powder for 12 weeks. The primary end point was the change from baseline (before supplement intake) to week 12 in the sum of scores and in each item score for the Mini-Mental State Examination (MMSE), HDS-R, Neuropsychiatric Inventory Nursing Home Version (NPI–NH), as well as each Z score, representing reduced regional cerebral blood flow relative to the normal reference data on n-isopropyl-4-iodoamphetamine hydrochloride (^123^I-IMP) single-photon emission computed tomography (SPECT), in subjects with cognitive impairment. We also investigated redox homeostasis in the brains of aged mice after they consumed a diet containing 0.5% quercetin.

## 2 Material and methods

### 2.1 Human clinical trial

#### 2.1.1 Human patients

Thirteen AD patients and six MCI patients aged 80.0 [75.5, 83.0] (median [IQR]) years were recruited for the study at two hospitals [Gifu University Hospital (the periods of recruitment and follow-up: from June 2018 to March 2021); Central Japan International Medical Center (from March 2020 to March 2021)] (female: n = 13; male: n = 6).

#### 2.1.2 Experimental design for clinical trial

**Fig. 1:**
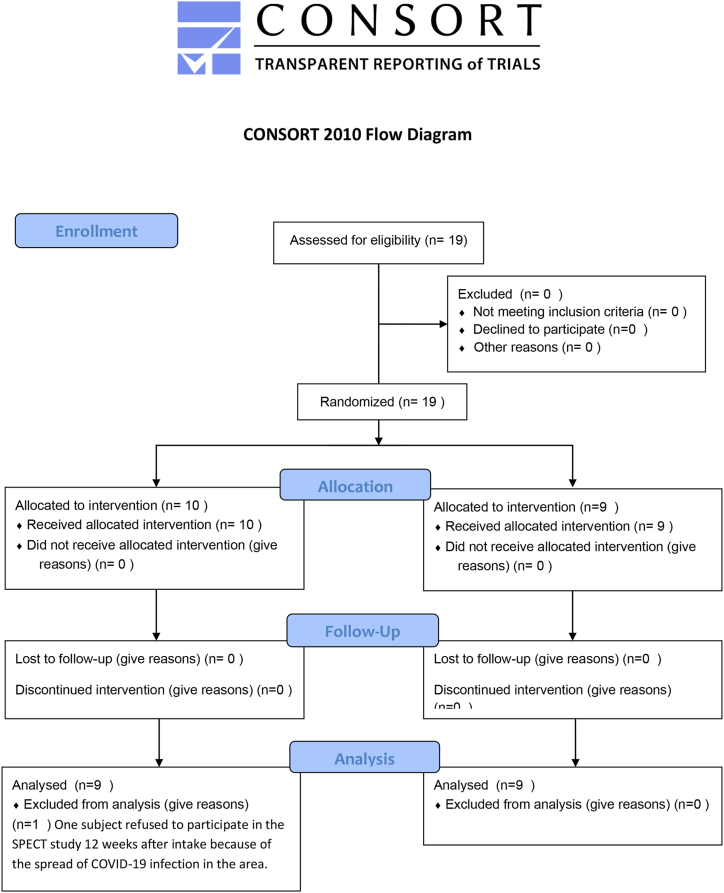
CONSORT flow diagram.

**Fig. 2:**
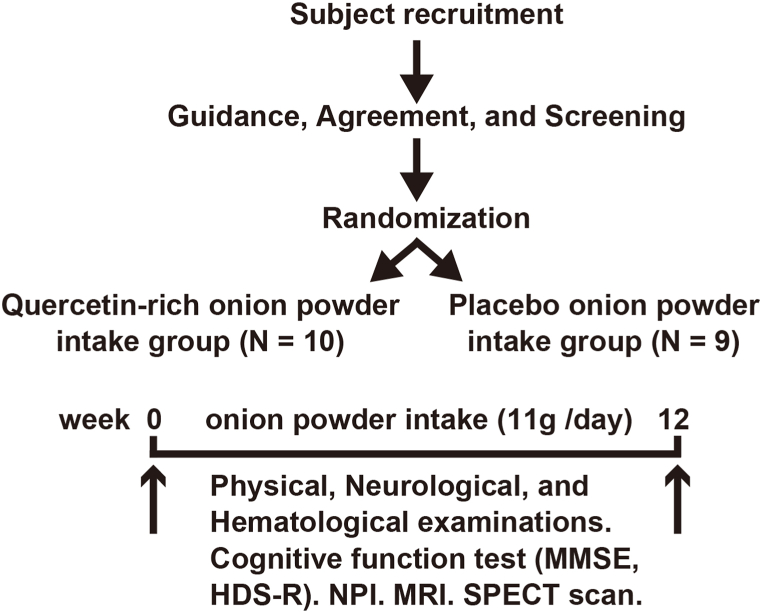
Schedule of clinical study. An age-matched randomized controlled study was performed. MMSE: Mini-Mental State Examination; HDS-R: Revised Hasegawa Dementia Scale; NPI-NH: Neuropsychiatric Inventory Nursing Home Version; MRI: magnetic resonance imaging; SPECT: single-photon emission computed tomography.

Patients were diagnosed with AD and MCI according to diagnostic guidelines for AD [19]. Briefly, the MMSE score was less than or equal to 23 in AD and 24–27 in MCI. Hippocampal atrophy was observed in AD, but not in MCI, assessed by MRI. Decreasing cerebral blood flow evaluated by SPECT using ^123^I-IMP injection was detected in regions of the parietal cortex, the precuneus, and the posterior cingulate cortex in AD but not in MCI. Impairment in one or more cognitive domains was evidenced in MCI, but those patients retained independence in functional abilities. Cognitive impairments caused by age-associated memory decline, polypharmacy, psychiatric conditions, and other neurological disorders were also differentially diagnosed from AD and MCI by physical, neurological, and hematological examinations. Therefore, we diagnosed thirteen subjects as having AD and six as having MCI (Core clinical criteria). We calculated sample size was twenty because two subjects were probably recruited every month during the period of this study. Blocked randomization was used: recruited subjects were sequentially assigned to one of the onion powder groups (block size = 1). T.N. enrolled participants and Y.H. assigned participants to interventions. Details about which onion powders contained detectable quercetin or placebo were concealed until all data were recorded, and information about the onion powders was opened after data were statistically analyzed. Only one author (Y·H.) had access to clinical information that could identify individual participants during the trial via electric medical records; however, no authors had access such an information after data collection. Patients consumed 11 g of onion powder containing quercetin glycosides equivalent to either 50 mg quercetin aglycone (quercetin-rich: “Sarasara Gold” onion) or a placebo containing less than the lower limit of detection of quercetin aglycone (placebo: white onion, with the lowest quercetin content) on a daily basis for 12 weeks. A randomized, double-blind, placebo-controlled study was conducted (parallel group, 1:1 allocation ratio) (Fig. 1, Fig. 2). Patients took the onion powder either directly or mixed with dishes such as miso soup.

The cognitive function of each group was assessed using the HDS-R, MMSE, and NPI-NH prior to intake of onion powder and after 12 weeks of intake. In addition to the neurological and cognitive assessments, a general physical examination was performed prior to the intake of onion powder and after 12 weeks of intake.

Brain MRI was performed prior to intake of onion powder. SPECT using ^123^I-IMP injection was performed prior to intake of onion powder and after 12 weeks of intake. A Z score was obtained based on the three-dimensional stereotactic surface projection (3D-SSP) technique [20], as follows: Z score = ([mean of normal reference data] – [individual subject value])/SD of normal reference data. Furthermore, a sentence of language component of the MMSE was evaluated by Text Mining Studio version 6.4 (NTT DATA Mathematical Systems Inc.).

### 2.2 Animal study

#### 2.2.1 Animal subjects

Thirty-two male C57BL/6 J mice aged 6 months were purchased from Japan SLC, Inc. (Hamamatsu, Japan).

#### 2.2.2 Experimental design for animal study

Mice were randomly divided into two groups. The first group (n = 17) received an AIN93G diet containing 20% casein (control), while the second (n = 15) received the same diet with the addition of 0.5% quercetin (quercetin) according to previous procedures [17]. The mice were housed in a temperature- and light-controlled room (24 °C; 12/12-h light/dark cycle), and each animal received water *ad libitum* and 4 g of food per day. The EPM test was conducted on a gray apparatus with two open arms (30 × 6 cm) and two closed arms (30 × 6 cm) meeting in a square central zone (6 × 6 cm); this maze was elevated 50 cm above the floor. Each mouse was acclimatized to the room for 1 h and exposed to the EPM for 1 min. The mice were placed in their home cages for 5 min. Then, the behavior of each mouse was monitored in the maze for 5 min using video recording software, an automated tracking system (SMART v3.0 software; Panlab, Barcelona, Spain) and a video camera (HDC-HS350; Panasonic; Osaka, Japan). Two weeks later, a forced swimming test (FST) was performed in a cylinder (diameter: 10 cm, height: 25 cm) containing water at 23 °C. The mice were acclimatized to the room for 1 h and then tested for 5 min. Global activity (cm^2^/s) was calculated using the SMART v3.0 tracking system (Panlab).

*In vivo* redox imaging was performed with an *in vivo* dynamic nuclear polarization magnetic resonance imaging (DNP-MRI) system (Keller, Japan redox Ltd., Fukuoka, Japan) with a 16 mT magnetic field for small animals. The electron paramagnetic resonance (EPR) irradiation frequency and MRI frequency were 458 MHz and 659 kHz, respectively. A circular, one-turn surface coil (inner diameter: 20 mm) was used to deliver the EPR irradiation for brain imaging. In *vivo* experiments, mice were anesthetized with 2% isoflurane and then secured in sternal recumbency on a special holder with adhesive skin tape. During the procedure, the body temperature of the mice was kept at 37 ± 1 °C with a heating pad. The holder was then placed in the resonator, and *in vivo* DNP-MRI imaging of the lower abdomen and hind limbs was started immediately after intramuscular administration of 150 mM 3-methoxycarbonyl-2,2,5,5-tetramethyl-pyrrolidine-1-oxyl (MC-PROXYL; 5 μL/g body weight). Pharmacokinetic DNP-MRI images were obtained every 16 s after injection. An *in vivo* redox map was obtained by taking the slope of the image intensity of each pixel in four pharmacokinetic images using a custom Excel macro program. The scanning conditions for the *in vivo* DNP-MRI experiment were as follows: power of EPR irradiation for the fast spin-echo sequence, 9 W; flip angle, 90°; repetition time (TR) × echo time (TE) × EPR irradiation time (TEPR), 500 × 37 × 300 m s; effective echo (TEeff), 20 msec, echo train length (ETL), 4; number of averages, 1; slice thickness, 100 mm (encompassing the full thickness of the mouse); phase-encoding steps, 32; field of view (FOV), 40 × 40 mm; and matrix size, 64 × 64 after reconstruction.

#### 2.2.3 Enzyme-linked immunosorbent assay (ELISA)

Dopamine release was measured with a Dopamine Research ELISA (ImmuSmol, Bordeaux, France). Brain tissue extracts were collected from the nucleus accumbens of animals that were fed the control chow diet (n = 8) or the quercetin-rich chow diet (n = 8) by using an extraction buffer [0.01 N HCl, 1 mM EDTA, 4 mM sodium metabisulfite, pH (7.0)] with Roche cOmplete Mini tablets (Roche, Mannheim, Germany) according to the manufacturer's protocol. BDNF levels were measured with a Mature BDNF Rapid ELISA Kit (Bisensis Pty Ltd., Thebarton, Australia). Additionally, brain tissue extracts were collected from the nucleus accumbens of animals that were fed the control chow diet (n = 8) or the quercetin-rich chow diet (n = 8) by using an acid extraction buffer [50 mM sodium acetate, 1 M NaCl, 0.1% Triton X-100, pH (4.0)] with Roche cOmplete Mini tablets (Roche, Mannheim, Germany) according to the manufacturer's protocol.

### 2.3 Sample size

In a previous pilot study conducted by Nakagawa et al. [17], the HDS-R memory recall component exhibited an increase of 3.2 ± 2.2 [mean ± standard deviation (SD)] points in the group consuming quercetin-rich onion powder, while the group consuming placebo onion powder showed no change (−0.4 ± 2.3 points) over the four-week observation period. These findings led us to hypothesize that the quercetin-rich onion powder group would demonstrate a greater difference in HDS-R scores compared to the placebo onion powder group over the twelve-week observation period.

Assuming a group difference of 3 and a pooled SD of 2.24 and a two-sided significance level of 5%, statistical power was calculated by Student's t-test to be 80.8%, therefore, subjects were recruited until the target number of 10 per group for this study was reached.

### 2.4 Statistical analysis

Continuous variables were summarized as the median and interquartile range (IQR), and differences between groups were evaluated using the Mann‒Whitney *U* test. Categorical variables were summarized using counts and proportions, and differences between groups were assessed using Fisher's exact test. Because the analysis in this study was exploratory, no adjustment was made for multiple comparisons. There were no missing values for any of the variables considered in human study. Statistical analysis was completed by September 25, 2022. A two-sided *p* < 0.05 was considered statistically significant. Statistical analyses were performed using SPSS Statistics 27 (IBM, Armonk, NY) and R version 4.1.1 (The R Project for Statistical Computing). Graphing of data was performed in Excel and RStudio Desktop (RStudio, Boston, MA) in mouse study. Statistical details are summarized in Table S1.

### 2.5 Ethics and institutional review board statement

We obtained informed consent from all participants by written consent form. All medical research and protocols involving human subjects were approved by the Gifu University Graduate School of Medicine Ethics Committee for Medical Research (approval number: 30–069) under the Ethical Guidelines for Medical and Health Research Involving Human Subjects as provided by the Ministry of Education, Culture, Sports, Science and Technology and Ministry of Health, Labour and Welfare of Japan. The work described was carried out in accordance with The Code of Ethics of the World Medical Association (Declaration of Helsinki) and we used the CONSORT reporting guidelines [21]. The clinical trial registration number is UMIN000033258.

All animal studies were approved by the Gifu University Graduate School of Medicine Animal Care and Use Committee (approval number: 2020–143) and were performed in accordance with the guidelines for experiments on animals as established by the Ministry of Education, Culture, Sports, Science and Technology of Japan. All animal experiments were performed according to the Animal Research: Reporting In Vivo Experiments (ARRIVE) guidelines.

## 3 Results

### 3.1 Human clinical trial

#### 3.1.1 Subjects were clinically diagnosed with MCI or AD and enrolled in the clinical study

A total of 19 subjects were included based on feasibility and randomly assigned: 9 to the placebo onion powder group and 10 to the quercetin-rich onion powder group. The demographic data of the subjects before the intake of each onion powder are shown in Table 1. Patients were diagnosed with AD and MCI according to diagnostic guidelines for AD [19]. Briefly, the 19 selected subjects were clinically diagnosed with MCI or AD on the basis of MMSE (less than or equal to 23 suggesting to AD, and 24–27 suggesting to MCI), HDS-R, MRI, and SPECT results, and were randomly divided into two groups. Although we followed the clinical criteria for the diagnosis of MCI, we did not test biomarkers reflecting amyloid-β (Aβ) such as cerebrospinal fluid (CSF) Aβ or Aβ positron-emission tomography (PET), which can be used to exclude other disorders affecting cognitive function.

**Table 1:** Characterization of subjects.

Variable	N	Overall N = 19^a^	Quercetin-rich onion powder group (Q) N = 10^a^	Placebo onion powder group (P) N = 9^a^	p-value^b^
age	19	80.0 (75.5, 83.0)	77.0 (74.2, 81.5)	82.0 (78.0, 83.0)	0.235
Sex	19				0.350
F		13 (68.4%)	8 (80.0%)	5 (55.6%)	
M		6 (31.6%)	2 (20.0%)	4 (44.4%)	
Diagnosis	19				0.628
AD		13 (68.4%)	6 (60.0%)	7 (77.8%)	
MCI		6 (31.6%)	4 (40.0%)	2 (22.2%)	
Cognitive assessment					
MMSE	19	23.0 (20.5, 26.0)	23.5 (19.2, 25.8)	23.0 (22.0, 26.0)	0.483
HDS_R	19	19.0 (16.0, 22.5)	19.0 (13.8, 19.8)	20.0 (17.0, 25.0)	0.269
NPI	19	4.0 (1.0, 7.0)	6.0 (4.0, 14.0)	1.0 (1.0, 3.0)	0.019

F: female; M: male; AD: Alzheimer's disease; MCI: mild cognitive impairment; N: number.

a Statistics presented: Median (IQR).

b Statistical tests performed: Wilcoxon rank sum test.

All 19 subjects completed the full study as scheduled (Fig. 1, Fig. 2) except for one subject in the quercetin-rich onion powder group, who refused to participate in the SPECT study 12 weeks after intake because of the spread of COVID-19 infection in the area. Therefore, we statistically analyzed a total of eighteen subjects in the SPECT study and nineteen subjects in the other studies. Hematological examination and other tests did not reveal any side effects (data not shown). This study was performed from June 2018 to March 2021.

#### 3.1.2 Changes in MMSE, HDS-R, and NPI scores in the two groups

**Fig. 3:**
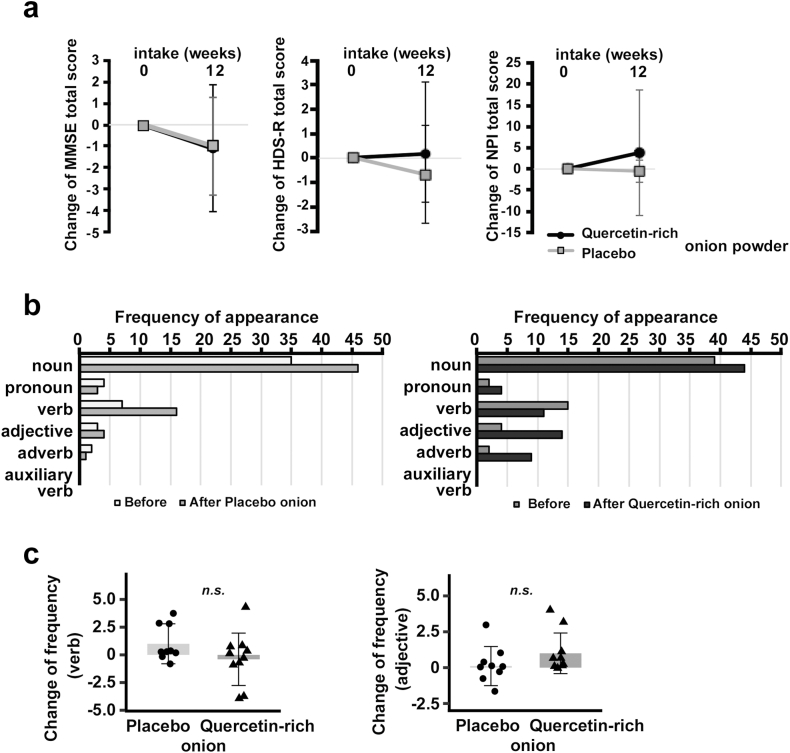
Changes in MMSE, HDS-R, and NPI scores. (**a**) Changes in MMSE (left panel), HDS-R (middle panel), and NPI scores (right panel). Quercetin-rich onion powder group (Q): n = 10; placebo onion powder group (P): n = 9. Values are shown as the mean ± SD. (**b**), Analyses of a sentence from the MMSE language test (left panel: placebo onion powder group; right panel: quercetin-rich onion powder group). All parts of speech were analyzed and counted before and after intake of each onion powder. (**c**) Changes in frequency (number of appearances after intake of onion powder minus number of appearances before intake) in subjects were not significantly different. (Left panel) Verbs. Median [IQR]: 0.00 [0.00, 3.00] (placebo); 0.00 [-1.00, 0.75] (quercetin-rich), *p* = 0.268 (Wilcoxon rank-sum test). (Right panel) Adjectives. Median [IQR]: 0.00 [0.00, 0.00] (placebo); 0.50 [0.00, 1.00] (quercetin-rich), *p* = 0.120 (Wilcoxon rank-sum test).

To evaluate the effect of the treatment on cognitive function, emotion, and mood, we administered MMSE, HDS-R, and NPI tests before and after the regimen of onion powder intake. The changes in total scores on the MMSE, HDS-R, and NPI were not significantly different between the quercetin-rich onion powder intake group and the placebo group from before intake to 12 weeks after intake of each onion powder (Fig. 3a). Since these tests examine several items, such as orientation, registration, attention and calculation, recall, language and praxis in MMSE, we analyzed the values for each item of the MMSE, HDS-R and NPI. Difference values were calculated as the score after 12 weeks of each onion powder minus the score prior to intake of onion powder. Only the changes in MMSE item 13 (writing) scores were significantly different between the two groups [*p* = 0.043; 95% confidence interval (0.031; 0.769)] (Table 2). Other item-specific values from the MMSE, HDS-R and NPI were not significantly different between the two groups (Table 2, and Table S2). Since sentence count, word count, number of adjectives, number of adverbs, and sentiment of response are used for depression assessment [22], we further analyzed a sentence obtained from MMSE item 13 b y Text Mining Studio version 6.4 (NTT DATA Mathematical Systems Inc.). Regarding the frequency of word use, subjects who took quercetin-rich onion powder wrote more adjectives and adverbs per sentence after intake (right panel in Fig. 3b). Furthermore, although we did not statistically compare the frequencies of specific adjectives or adverbs (Fig. 3c), subjects after intake of quercetin-rich onion powder used descriptors such as “happy”, “brightly”, “cool”, “fun”, “fine”, “ashamed”, “hot”, “well”, “delighted”, and “hard”, while subjects in placebo onion powder used descriptors such as “lonely”, “many”, “quickly”, and “fine”.

**Table 2:** Changes in MMSE, HDS-R, and NPI scores.

Variable	N	Overall, N = 19^a^	Q, N = 10^a^	P, N = 9^a^	p-value^b^
MMSE	19	−1.0 (−3.0, 0.0)	−2.0 (−3.0, 1.5)	−1.0 (−2.0, 0.0)	0.773
MMSE1	19	−1.0 (−1.0, 0.0)	0.0 (−1.8, 0.0)	−1.0 (−1.0, 0.0)	0.704
MMSE2	19	0.0 (−0.5, 0.0)	0.0 (−1.0, 0.0)	0.0 (0.0, 0.0)	0.181
MMSE3	19	0.0 (0.0, 0.0)	0.0 (0.0, 0.0)	0.0 (0.0, 0.0)	0.651
MMSE4	19	0.0 (0.0, 0.0)	0.0 (0.0, 0.0)	0.0 (0.0, 0.0)	0.193
MMSE5	19	0.0 (0.0, 0.0)	0.0 (0.0, 0.0)	0.0 (0.0, 0.0)	0.359
MMSE6	19	0.0 (0.0, 0.0)	0.0 (0.0, 0.0)	0.0 (0.0, 0.0)	>0.999
MMSE7	19	0.0 (−1.0, 0.0)	−0.5 (−1.0, 0.0)	0.0 (−1.0, 0.0)	0.966
MMSE8	19	0.0 (0.0, 0.0)	0.0 (0.0, 0.0)	0.0 (0.0, 1.0)	0.062
MMSE9	19	0.0 (0.0, 0.0)	0.0 (0.0, 0.0)	0.0 (0.0, 0.0)	>0.999
MMSE10	19	0.0 (0.0, 0.0)	0.0 (0.0, 0.0)	0.0 (0.0, 0.0)	0.359
MMSE11	19	0.0 (0.0, 0.0)	0.0 (0.0, 0.0)	0.0 (0.0, 0.0)	0.343
MMSE12	19	0.0 (0.0, 0.0)	0.0 (0.0, 0.0)	0.0 (0.0, 0.0)	>0.999
MMSE13	19	0.0 (0.0, 0.0)	0.0 (0.0, 1.0)	0.0 (0.0, 0.0)	0.043
MMSE14	19	0.0 (−1.0, 0.0)	0.0 (−0.8, 0.0)	0.0 (−1.0, 0.0)	0.963
HDS_R	19	0.0 (−2.5, 1.5)	1.0 (−1.8, 1.8)	0.0 (−3.0, 0.0)	0.510
HDS_R1	19	0.0 (0.0, 0.0)	0.0 (0.0, 0.0)	0.0 (0.0, 0.0)	>0.999
HDS_R2	19	−1.0 (−1.0, 0.0)	0.0 (−1.8, 0.0)	−1.0 (−1.0, 0.0)	0.704
HDS_R3	19	0.0 (0.0, 0.0)	0.0 (0.0, 0.0)	0.0 (0.0, 0.0)	0.519
HDS_R4	19	0.0 (0.0, 0.0)	0.0 (0.0, 0.0)	0.0 (0.0, 0.0)	>0.999
HDS_R5	19	0.0 (−0.5, 0.0)	0.0 (0.0, 0.0)	0.0 (−1.0, 0.0)	0.272
HDS_R6	19	0.0 (0.0, 1.0)	0.0 (0.0, 0.8)	0.0 (0.0, 1.0)	0.462
HDS_R7	19	0.0 (0.0, 0.0)	0.0 (0.0, 0.0)	0.0 (0.0, 1.0)	0.124
HDS_R8	19	0.0 (−0.5, 1.0)	0.0 (−0.8, 0.8)	0.0 (0.0, 1.0)	0.734
HDS_R9	19	0.0 (0.0, 0.0)	0.0 (0.0, 0.8)	0.0 (0.0, 0.0)	0.124
NPI	19	0.0 (−1.0, 1.5)	0.5 (−2.2, 9.0)	0.0 (0.0, 1.0)	0.589

Values for each group are calculated as the test score after 12 weeks of onion powder intake minus the score prior to intake. Asterisks (*) indicate results that are considered statistically significant. MMSE (total score); HDS-R (total score); NPI (total score). The MMSE score included 14 items [1: date orientation; 2: seasonal orientation; 3: place orientation (prefecture, city, hospital name); 4: place orientation (floor number); 5: place orientation (state); 6: registration; 7: calculation; 8: recall; 9: naming; 10: repetition; 11: three-stage command; 12: reading; 13: writing; 14: copying]. The HDS-R score involved 9 items [1: age; 2: date orientation; 3: place orientation (hospital name); 4: registration; 5: calculation; 6: backward counting; 7: recall after registration (three unrelated terms); 8: recall after presentation of five unrelated objects; 9: vegetable naming].

a Statistics presented: Median (IQR).

b Statistical tests performed: Wilcoxon rank sum test.

#### 3.1.3 Changes in the regional Z scores of SPECT images between the two groups

**Fig. 4:**
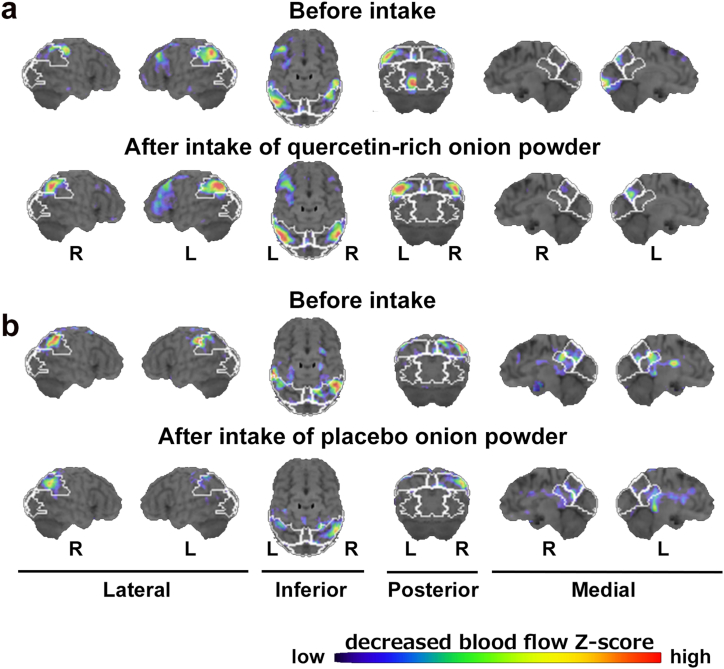
**Representative Z-score images from the**^**123**^**I-IMP SPECT study before and after 12 weeks of intake of each onion powder.** Z-score images in subjects before and after intake of quercetin-rich onion powder (**a**) and placebo onion powder (**b**). A higher Z score indicates lower regional cerebral blood flow (rCBF). The Z-score scale is indicated by the color gradient from blue to red (lower rCBF). R and L refer to the right side and the left side, respectively.

Using Z scores obtained through 3D-SSP, it is possible to evaluate dysfunctional brain regions and differentiate between AD and Lewy body disease [23]. Therefore, we used Z scores to assess the effect of quercetin-rich onion powder on brain function. Fig. 4 shows representative Z score images from the ^123^I-IMP SPECT study before and after 12 weeks of intake of each onion powder. Using the SPECT images, we compared the Z score of each region between the quercetin-rich onion powder group and the placebo group before and after the intake of onion powder. These pre-to post-intake changes in regional Z scores for 10 regions (parietal, precuneus, cingulate, posterior lateral or medial areas in right or left) were not significantly different between the quercetin-rich onion powder group and the placebo group (Table 3).

**Table 3:** Changes in the regional Z scores of^123^I-IMP SPECT images.

Area	N	Overall, N = 19^a^	Q, N = 10^a^	P, N = 9^a^	p-value^b^
Parietal (R)	18	0.0 (−0.9, 0.9)	0.0 (−0.6, 0.9)	−0.1 (−0.9, 0.7)	0.666
Parietal (L)	18	0.7 (−0.4, 1.0)	0.6 (0.0, 0.7)	0.7 (−0.7, 1.3)	>0.999
Precuneus (R)	18	0.3 (−0.6, 1.0)	0.2 (−0.1, 1.1)	0.4 (−1.6, 0.7)	0.489
Precuneus (L)	18	0.1 (−0.3, 1.0)	0.7 (0.0, 2.9)	−0.1 (−1.2, 0.3)	0.077
Cingualate (R)	18	−0.1 (−0.8, 0.7)	0.7 (−0.4, 0.7)	−0.3 (−1.4, 0.0)	0.216
Cingulate (L)	18	−0.3 (−0.8, 0.5)	−0.2 (−0.6, 0.7)	−0.3 (−1.3, 0.2)	0.310
Posterior.lateral (R)	18	0.0 (−0.3, 0.0)	0.0 (−0.3, 0.1)	0.0 (−0.3, 0.0)	0.860
Posterior.lateral (L)	18	0.0 (−0.4, 0.1)	0.0 (−0.5, 0.0)	0.0 (−0.3, 0.2)	0.330
Posterior.medial (R)	18	0.0 (−0.2, 0.1)	0.0 (−0.2, 0.1)	0.0 (−0.1, 0.1)	0.658
Posterior.medial (L)	18	0.0 (−0.3, 0.2)	0.0 (−0.2, 0.2)	0.0 (−0.3, 0.0)	0.627

Values are calculated by regional Z scores after 12 weeks of onion powder intake minus the score prior to intake. R and L refer to the right side and the left side, respectively.

a Statistics presented: Median (IQR).

b Statistical tests performed: Wilcoxon rank sum test.

### 3.2 Animal study

#### 3.2.1 Quercetin altered the behavior of aged mice in the EPM test

**Fig. 5:**
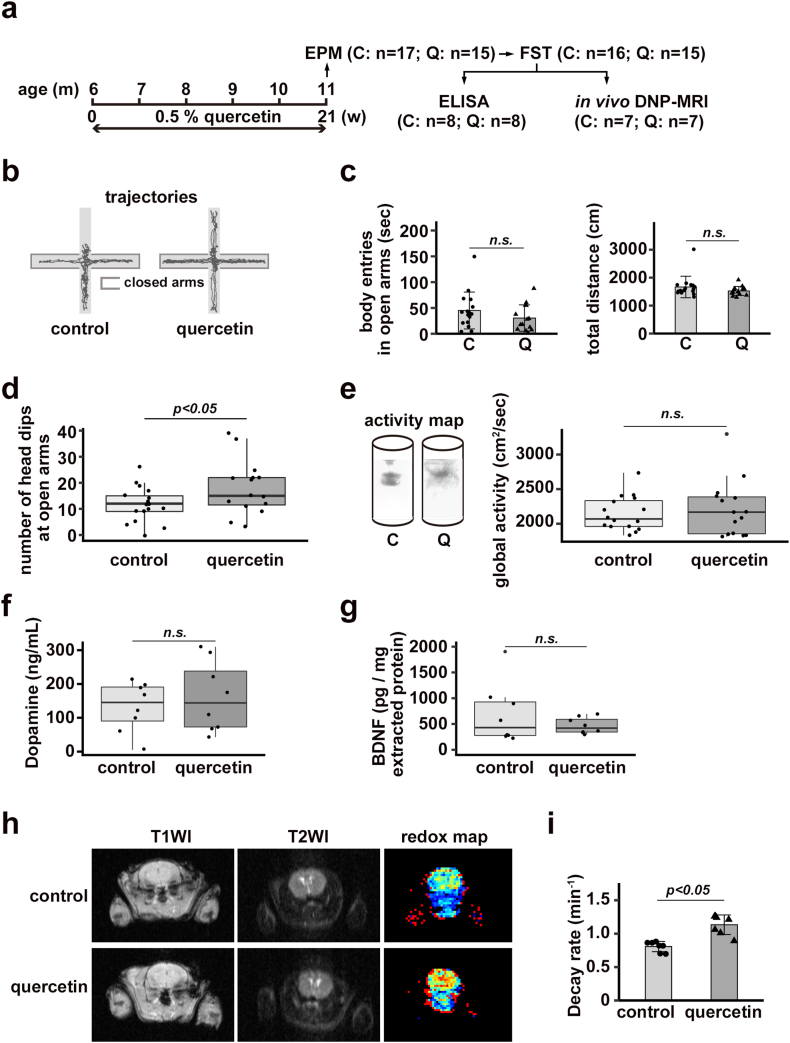
A-g Aged wild-type mice that were fed a quercetin-supplemented chow diet exhibited an increase in head dipping from the open arms of the EPM. (**a**) Experimental schedule. (**b-d**) Representative trajectories of mice walking in the open and closed arms of the EPM (b). The time spent in the open arms (left panel in c) and the total distance traveled in the maze (right panel in c) were assessed in mice that received a control chow diet (n = 17) and a quercetin-enriched chow diet (n = 15) (c). The number of head dips from the open arms was assessed in mice that were fed the quercetin-rich chow diet (n = 15) compared to mice that were fed the control chow diet (n = 17) (d). (**e**) Global activity of mice in the FST was assessed in mice that were fed the control chow diet (n = 16) and the quercetin-rich chow diet (n = 15). (**f, g**) Dopamine release (f) and BDNF production (g), as measured by ELISAs, in the nucleus accumbens of mice that were fed the control chow diet (n = 8) and the quercetin-rich chow diet (n = 8)**. h-i *in vivo* mouse brain imaging.** (**h, i**) Representative MRI and *in vivo* brain DNP-MRI of mice that received the control chow diet (n = 7) and the quercetin-rich chow diet (n = 7) (h). The decay rates of pixels in the DNP-MRI brain scans were calculated and visualized as a redox map (right panels in h). The decay rates (min^−1^) in each pixel of the DNP MRI scans were calculated according to the slope of the image intensity in the region of interest corresponding to the MC-PROXYL enhancement (i; n = 7, 7; *p* < 0.05). T1WI: time 1–weighted imaging; T2WI: time 2–weighted imaging.

We examined whether quercetin affects anxiety- and depression-like behavior in aged wild-type mice (Fig. 5a–e). First, we examined the EPM test for anxiety-like behavior (Fig. 5b–d). The two groups of mice moved similarly (right graph in Fig. 5c) and entered the open arms (left graph in Fig. 5c). We found that the number of head dips from the open arms was significantly increased in mice that received the quercetin-rich chow diet compared to mice that received the control chow diet (Fig. 5d), which is validated as a measure of anxiety [24]. Next, we used the FST. Global activity in the FST was not different between groups (Fig. 5e). Since Bastioli et al. recently demonstrated that exercise enhances dopamine release in the striatum and nucleus accumbens (NAc) via BDNF [25], we examined the levels of dopamine and BDNF in the NAc of mice by ELISA (Fig. 5f and g). We did not observe a significant difference (Fig. 5f). We also did not observe an intergroup difference in the BDNF context of the NAc (Fig. 5g). More experiments are needed to explore the involvement of these molecules in anxiety- and depression-like behavior.

#### 3.2.2 Quercetin improved redox homeostasis in the mouse brain

Quercetin has antioxidant activity, producing beneficial effects in several diseases [26]. Since oxidation of thiol/disulfide systems increases with age and redox status deteriorates during aging in humans [27], we investigated redox activity in the mouse brain using *in vivo* DNP-MRI (Fig. 5h–i). Interestingly, we found that redox activity, evaluated by the decay rate of the blood–brain barrier (BBB)-permeable probe MC-PROXYL [28], was significantly increased in the brains of mice that received the quercetin-rich chow diet compared with mice that received the control chow diet (Fig. 5i). We did not observe any change in brain morphology upon a regular anatomical analysis of the MR images.

## 4 Discussion

In this study, we found that subjects who took quercetin-rich onion powder wrote more adjectives and adverbs per sentence after onion intake than before on the MMSE language test compared to subjects who took placebo onion powder. We also demonstrated that in mice that received the quercetin-rich chow diet, the number of head dips from the open arms was significantly increased in the elevated plus maze (EPM) and redox activity was significantly increased in the brain.

Quercetin, an abundant natural dietary flavonoid, is recognized as a promising neuroprotective compound that acts through several mechanisms, such as anti-inflammatory, antidiabetic, antiproliferative, antiviral, and antioxidant effects [13]. Interestingly, an anxiolytic/antidepressant-like action of quercetin has been demonstrated in animal models of depression [15]. However, the effect of quercetin-rich onion on the emotional condition of people living with cognitive impairment remains to be determined. Recently, it was shown that continuous intake of quercetin-rich onion suppresses cognitive decline in aged people by improving their emotional condition [18]. In the present randomized, double-blind, placebo-controlled study, we demonstrated that subjects with MCI or AD cognitive impairment wrote more adjectives and adverbs per sentence in the MMSE language test after a regimen of quercetin-rich onion intake than before intake. However, a difference in regional cerebral blood flow on ^123^I-IMP SPECT was not observed between the quercetin-rich and placebo onion powder groups.

Depression and anxiety overlap, and it has been hypothesized that neural dysregulation in depression may be mediated by the stress system (hippocampus and hypothalamic–pituitary–adrenal (HPA) axis) and the reward system (ventral tegmental area (VTA)-NAc pathway) [29,30]. BDNF plays a critical role in depression and anxiety, and antidepressants exert their effects by activating BDNF-tropomyosin receptor kinase B (TrkB) signaling [30]. It has also been indicated that BDNF regulates exercise-induced dopamine release, underpinning the effects of exercise in dopamine-related mood disorders [25]. In our experiments, the number of head dips from the open arms of the EPM was significantly increased in mice that received the quercetin-rich chow diet Although we did not observe a difference in the concentrations of dopamine or BDNF between the NAcs of control and quercetin-supplemented mice, more experiments are needed to explore the role of quercetin in dopamine release and *BDNF* mRNA expression in light of two prior findings. First, the levels of dopamine are increased by quercetin in the 1-methyl-4-phenyl-1,2,3,6-tetrahydropyridine (MPTP)-induced mouse model of Parkinson's disease (PD) [31]. Second, quercetin-3-*o*-glucuronide (Q3G), a major metabolite that accumulates in the brain and other tissues after oral administration of quercetin [32], increases *BDNF* mRNA expression in human embryonic neural stem cells (NSCs) [33].

I*n vivo* DNP-MRI measuring redox activity in the mouse brain showed that redox activity was significantly increased in the brain tissue of mice that received the quercetin-rich chow diet compared with mice that received the control chow diet. BBB-permeable MC-PROXYL is a nitroxyl radical that acts biologically as a superoxide dismutase mimic, antioxidant, and spin probe; therefore, its decay rate indicates the redox status of the brain. A high concentration of reactive oxygen species (ROS) is suggested to mediate oxygen toxicity and thereby disrupt redox signaling, while a low concentration of ROS is reported to shift redox signaling toward cell growth and differentiation, for example, in stem cells [27,34]. Since redox status deteriorates during aging in humans [27], we demonstrated that quercetin may prevent deterioration of redox homeostasis. In the ER, ROS are produced from protein oxidation by ER oxidoreductin-1 (ERO1) and NADPH oxidase (NOX) through disulfide bond generation catalyzed by protein disulfide isomerase (PDI) during protein folding [35]. The accumulation of misfolded proteins in the ER stimulates ER stress signaling to reduce the burden of misfolded proteins; however, persistent ER stress induces ROS production that leads to an imbalance in redox homeostasis [35,36], while an imbalance in redox homeostasis elicits ER stress [[37], [38], [39]], demonstrating that redox and ER stress control each other [40].

We assessed cognition and emotion in patients using the MMSE, HDS-R, and NPI-NH. Interestingly, in the MMSE, subjects who received quercetin-rich onion wrote more adjectives and adverbs per sentence after supplement intake than before intake (“MMSE13”). Text analysis allows researchers to assess moods, emotions, and depression [41]. Early depression detection is important for interventions; for this purpose, Guohou S. et al. Developed automatic depression detection using machine learning [22]. In the category of verbal features, they listed several textual subcategories, including sentence count, word count, number of adjectives, number of adverbs, and sentiment of response, as useful variables for depression assessment. Using text mining software, we found that subjects with cognitive impairment used more adjectives and adverbs per sentence after taking quercetin-rich onion powder than before taking the supplement. The evidence and results suggested that quercetin may influence the emotional condition of people living with cognitive impairment, which it may accomplish by controlling ER stress and maintaining redox homeostasis, although more study is needed to explore the precise mechanism of action of quercetin.

## 5 Conclusions

In this randomized, double-blind, placebo-controlled study, we did not identify any effect of quercetin-rich onion intake on regional cerebral blood flow or measures of cognitive performance (MMSE and HDS-R). However, on the MMSE language test, subjects who took quercetin-rich onion powder wrote more adjectives and adverbs per sentence after onion intake than before, whereas subjects who took low-quercetin placebo onion powder showed no such change. Several limitations are worth noting. Although we calculated sample size was twenty (each 10 patients/group) during the period of this study, it was small. Subjects wrote sentences as part of the MMSE test in the consultation room at the hospital and all tests including MMSE, neurological, physical, and hematological examinations on the same day; however, MR and SPECT images were taken within one week. Future research may clarify these concerns, and the effects of quercetin-rich onion on emotional condition may be assessed in subjects with MCI due to AD.

## Author contribution statement

Yuichi Hayashi: Performed the experiments; Analyzed and interpreted the data; Contributed reagents, materials, analysis tools or data; Wrote the paper.

Fuminori Hyodo, Tana, Kiyomi Nakagawa, Takuma Ishihara, Masayuki Matsuo, Takayoshi Shimohata: Performed the experiments; Analyzed and interpreted the data; Wrote the paper.

Jun Nishihira: Performed the experiments; Analyzed and interpreted the data.

Masuko Kobori: Conceived and designed the experiments; Wrote the paper.

Toshiyuki Nakagawa: Conceived and designed the experiments; Performed the experiments; Analyzed and interpreted the data; Contributed reagents, materials, analysis tools or data; Wrote the paper.

## Data availability statement

Data will be made available on request.

## Declaration of competing interest

The authors declare that they have no known competing financial interests or personal relationships that could have appeared to influence the work reported in this paper.
