# Supplementary material for: Continuous intake of quercetin-rich onion powder may improve emotion but not regional cerebral blood flow in subjects with cognitive impairment
Source: Heliyon. 2023 Jul 19;9(8):e18401. doi: 10.1016/j.heliyon.2023.e18401 (PMC10391933; doi:10.1016/j.heliyon.2023.e18401)
Supplement: Multimedia component 1 [file mmc1.docx]

| **S1 Table-Statistical summary** | | | | | |
| --- | --- | --- | --- | --- | --- |
| **Fig., Table** | **Experiment** | **Groups** | **Test used** | **Values** | **P value** |
| Fig.3a | MMSE | the continuous intake of quercetin-rich onion powder (n = 10) and placebo onion powder (n = 9) for 12 weeks | Student’s t test (two-tailed) | t17 = 0.082 | 0.936 |
|  | HDS-R |  |  | t17 = 0.743 | 0.468 |
|  | NPI |  |  | t17 = 0.882 | 0.390 |
| Fig. 3c | change of frequency (verb) | the continuous intake of quercetin-rich onion powder and placebo onion powder for 12 weeks |  | t17 = 1.437 | 0.169 |
|  | change of frequency (adjective) |  |  | t17 = 1.391 | 0.182 |
| Fig. 5c | EPM (body entry) | a control chow diet (n = 16) and  a quercetin-enriched chow diet (n = 14) |  | t28 = 1.273 | 0.213 |
|  | EPM (total distance) |  |  | t28 = 1.294 | 0.206 |
| Fig. 5d | EPM (head dip) | a control chow diet (n = 17) and  a quercetin-enriched chow diet (n = 15) |  | t30 = 2.106 | 0.044 |
| Fig. 5e | FST (global activity) | a control chow diet (n = 16) and  a quercetin-enriched chow diet (n = 15) |  | t29 = 0.542 | 0.592 |
| Fig. 5f | ELISA (dopamine) | a control chow diet (n = 8) and  a quercetin-enriched chow diet (n = 8) |  | t14 = 0.664 | 0.518 |
| Fig. 5g | ELISA (BDNF) | a control chow diet (n = 8) and  a quercetin-enriched chow diet (n = 8) |  | t14 = 1.007 | 0.331 |
| Fig. 5i | DNP MRI | a control chow diet (n = 7) and  a quercetin-enriched chow diet (n = 7) |  | t12 = 5.235 | 0.000 |
| Table 1 | a randomized, double-blind, placebo-controlled study in people living with cognitive impairment (AD, MCI) | the continuous intake of quercetin-rich onion powder and placebo onion powder for 12 weeks | wilcoxon rank-sum test |  | table 1 |
| Table 2 | MMSE, HDS-R, NPI | the continuous intake of quercetin-rich onion powder and placebo onion powder for 12 weeks | wilcoxon rank-sum test |  | table 2 |
| Table 3 | ^123^IMP-SPECT | the continuous intake of quercetin-rich onion powder and placebo onion powder for 12 weeks | wilcoxon rank-sum test |  | table 3 |
| S2. Table | NPI items | the continuous intake of quercetin-rich onion powder and placebo onion powder for 12 weeks | wilcoxon rank-sum test |  | S2 Table |
