# Supplementary material for: Continuous intake of quercetin-rich onion powder may improve emotion but not regional cerebral blood flow in subjects with cognitive impairment
Source: Heliyon. 2023 Jul 19;9(8):e18401. doi: 10.1016/j.heliyon.2023.e18401 (PMC10391933; doi:10.1016/j.heliyon.2023.e18401)
Supplement: Multimedia component 2 [file mmc2.docx]

**S2 Table. Changes of score in NPI items**

| Variable | N | Overall, N = 19^1^ | P, N = 10^1^ | Q, N = 9^1^ | p-value^2^ |
| --- | --- | --- | --- | --- | --- |
| total score | 19 | 0.0 (-1.0, 1.5) | 0.5 (-2.2, 9.0) | 0.0 (0.0, 1.0) | 0.589 |
| Delusions | 19 | 0.0 (0.0, 0.0) | 0.0 (0.0, 0.0) | 0.0 (0.0, 0.0) | 0.193 |
| Hallucinations | 19 | 0.0 (0.0, 0.0) | 0.0 (0.0, 0.0) | 0.0 (0.0, 0.0) | >0.999 |
| Agitation  /Aggression | 19 | 0.0 (0.0, 0.0) | 0.0 (0.0, 1.8) | 0.0 (0.0, 0.0) | 0.125 |
| Depression  /Dysphoria | 19 | 0.0 (0.0, 0.0) | 0.0 (0.0, 0.8) | 0.0 (0.0, 0.0) | 0.151 |
| Anxiety | 19 | 0.0 (0.0, 1.5) | 0.0 (0.0, 2.5) | 0.0 (0.0, 1.0) | 0.659 |
| Elation  /Euphoria | 19 | 0.0 (0.0, 0.0) | 0.0 (0.0, 0.0) | 0.0 (0.0, 0.0) | >0.999 |
| Apathy  /Indifference | 19 | 0.0 (0.0, 1.0) | 0.0 (-0.8, 1.5) | 0.0 (0.0, 1.0) | 0.931 |
| Disinhibition | 19 | 0.0 (0.0, 0.0) | 0.0 (0.0, 0.0) | 0.0 (0.0, 0.0) | >0.999 |
| Irritability  /Lability | 19 | 0.0 (0.0, 0.0) | 0.0 (-1.5, 3.5) | 0.0 (0.0, 0.0) | 0.706 |
| Aberrent Motor Behavior | 19 | 0.0 (0.0, 0.0) | 0.0 (-0.8, 0.0) | 0.0 (0.0, 0.0) | 0.456 |

| ^1^Statistics presented: Median (IQR) |
| --- |
| ^2^Statistical tests performed: Wilcoxon rank sum test |
